# The predictive value of the Kampala Trauma Score (KTS) in the outcome of multi-traumatic patients compared to the estimated Injury Severity Score (eISS)

**DOI:** 10.1186/s12873-024-00989-w

**Published:** 2024-05-14

**Authors:** Zahra Hakimzadeh, Samad Shams Vahdati, Alireza Ala, Farzad Rahmani, Rouzbeh Rajaei Ghafouri, Mehran Jaberinezhad

**Affiliations:** 1 Emergency and Trauma Care Research Center, Tabriz University of Medical Sciences, Tabriz, Iran; 2 Clinical Research Development Unit of Tabriz Valiasr Hospital, Tabriz University of Medical Sciences, Tabriz, Iran

**Keywords:** Kampala trauma score, Estimated injury severity score, Trauma, Triage, Injury scoring system

## Abstract

**Purpose:**

The classification of trauma patients in emergency settings is a constant challenge for physicians. However, the Injury Severity Score (ISS) is widely used in developed countries, it may be difficult to perform it in low- and middle-income countries (LMIC). As a result, the ISS was calculated using an estimated methodology that has been described and validated in a high-income country previously. In addition, a simple scoring tool called the Kampala Trauma Score (KTS) was developed recently. The aim of this study was to compare the diagnostic accuracy of KTS and estimated ISS (eISS) in order to achieve a valid and efficient scoring system in our resource-limited setting.

**Methods:**

We conducted a cross-sectional study between December 2020 and March 2021 among the multi-trauma patients who presented at the emergency department of Imam Reza hospital, Tabriz, Iran. After obtaining informed consent, all data including age, sex, mechanism of injury, GCS, KTS, eISS, final outcome (including death, morbidity, or discharge), and length of hospital stay were collected and entered into SPSS version 27.0 and analyzed.

**Results:**

381 multi-trauma patients participated in the study. The area under the curve for prediction of mortality (AUC) for KTS was 0.923 (95%CI: 0.888–0.958) and for eISS was 0.910 (95% CI: 0.877–0.944). For the mortality, comparing the AUCs by the Delong test, the difference between areas was not statistically significant (p value = 0.356). The diagnostic odds ratio (DOR) for the prediction of mortality KTS and eISS were 28.27 and 32.00, respectively.

**Conclusion:**

In our study population, the KTS has similar accuracy in predicting the mortality of multi-trauma patients compared to the eISS.

**Supplementary Information:**

The online version contains supplementary material available at 10.1186/s12873-024-00989-w.

## Introduction

Traumatic injuries are considered a major cause of mortality and morbidity worldwide and based on statistics, 90% of trauma deaths occur in low- and middle-income countries (LMIC) [1, 2]. The World Health Organization estimates that by 2030, trauma deaths will increase by 40% [3]. In developing countries, trauma is the leading cause of years of life lost (YLL) and the second leading cause of death in all age groups in Iran [3, 4]. 

In high-income countries (HIC), improvements to the quality and processes of trauma care have led to a significant reduction in mortality and disability [5]. The use of standardized trauma scoring systems is essential to appropriate triage, identifying the impact of injury, and assessing the quality of trauma care and hospital performance [6–8]. It also enables physicians and scientists to quantify injury severity and evaluate clinical and economic consequences [9]. In this regard, more investments in study and development are required to find efficient injury managing strategies as disability becomes a greater component of disease burden and health expenditure [10]. 

The Injury Severity Score (ISS), developed by Baker et al. in 1974, is the most widely used and verified trauma score [11]. The ISS is composed of the three highest Abbreviated Injury Scale (AIS) scores for the three most severely injured body regions, with a range from 1 (minor severity) to 75 (maximum severity) [12–14]. However, the ISS performs well in industrialized countries, it might be challenging to calculate and use in low-income countries where trained manpower, comprehensive medical records, and advanced diagnostic studies (radiography and autopsy) often are not available [6, 15]. This anatomical score is typically calculated upon patient discharge or based on the patient’s most recent condition and has thus been applied to retrospective assessment [16]. In addition, retrospective administrative data often do not provide details on possible risk factors, treatment costs, modalities, or patient outcomes [17]. As a result, calculating ISS was performed in an estimated method (eISS) as previously described [16, 18–20]. In LMIC settings, AIS frequently was generated based on the clinical evaluation conducted in the emergency department, then eISS was calculated based on the AIS for each patient [17]. This methodology has already been described and validated in a high-income country, therefore, the term estimated ISS is used in this report [17]. 

Kampala Trauma Score (KTS) was derived by Kobusingye et al. as a simple and feasible trauma scoring tool that can be utilized in front-line triage in resource-limited settings [15, 21]. KTS assesses injury primarily based on physiological factors rather than anatomical ones and relies on the patient’s age, systolic blood pressure, respiratory rate, neurologic status, and the number of serious injuries with a range from 5 to 16, with lower scores representing more severe injury [20]. 

Since the KTS needs a single algorithm to be applied to all age groups and requires fewer variables for calculation than other triage tools, it may be a more easy-to-use tool than others [22]. Also, it eliminates the need for a retrospective examination of injuries, and reduces the associated manpower and financial resource needs which are limited in LMICs settings. Thus, an easy to collect scoring system like the KTS is more practical for front-line health management in emergency medicine [23, 24]. A previous study revealed that the KTS had clinically substantial capacity to predict hospital admission needs, regardless of whether serious injury was determined by physician opinion or estimated AIS score, this makes the KTS even more simple and applicable in these settings [22]. In addition, incomplete imaging in LMICs precludes the use of ISS in the casualty department of LMICs; while, physiological scores like the KTS will be the practical score to use in casualty departments [24]. 

Although multiple scoring methods for injury severity have been developed and validated in recent decades, few studies have investigated the effectiveness of different trauma scoring systems in developing countries. An accurate selection of trauma scoring systems in these settings, which have much higher rates of injury burden, is crucial.

Although KTS has been shown to have the potential to replace other scoring systems, the literature supporting the utility of this tool over others is currently limited in Iran. An overcrowded emergency room results in a number of issues including staff tiredness, prolonged waiting time, poor quality of patient management, treatment disruption, and inadequate privacy. Due to limited health care resources in such settings, personal and systematic errors in applying the complex algorithm are inevitable and patient care could not be provided without conservative resource allocation [25]. Consequently, in order to accurately determine the severity of trauma and extensively evaluate the outcomes of trauma centers, it would be prudent to apply an appropriate and simple trauma scoring system [26]. 

This study aimed to evaluate the predictive value of KTS in the patient’s outcome as well as the length of hospital stay (LOS) as an important criterion for evaluating trauma care compared to estimated ISS.

## Patients and method

We conducted a cross-sectional study between December 2020 and March 2021 among the multi-trauma patients who presented at the emergency department of Imam Reza hospital, which is considered the primary academic trauma center of the East Azarbaijan province, and all trauma patients found by EMS are brought to this center located in Tabriz, Iran. The inclusion criterion was all multi-trauma patients (the presence of injury to more than one body area or system) of any age presenting to the emergency department [27]. 

Patients who did not consent to participate in this study for any reason, or who had a history of alcohol consumption or any drugs that altered the level of consciousness (due to control of the confounder variable in evaluation of neurological status), as well as patients who were transferred from other low-level hospitals (because of the possibility of modifying physiologic derangements), and also patients with missing data on any necessary components were excluded in our analysis.

Data were collected in two stages by one research assistant. The research assistant was trained in a two-day course for AIS calculation and trauma patient management under the supervision of ATLS® instructor. In the first stage, after obtaining informed written consent, data regarding the patient’s age, sex, mechanism of injury (car crash, motorcycle crash, bicycle crash, car to pedestrian, fall), vital signs (systolic blood pressure and respiratory rate), AVPU neurologic status, and level of consciousness (GCS value) were collected during the patient’s initial presentation to the emergency department. Then, the same research assistant completed the collection of the AIS needed to calculate the Kampala Trauma Score (KTS) and estimated Injury Severity Score (eISS) based on the secondary survey and the initial diagnosis tests including FAST and radiographs Imaging studies were performed only for patients who needed urgent examination based on the emergency resident physician’s opinion. The number of serious injuries used in the KTS for each patient was determined based on a list of primary diagnoses in the emergency department and derived from an AIS cutoff of two or more to be considered a serious injury [19]. The estimated ISS was calculated manually based on the methodology introduced in the AIS-80 and the same algorithm as ISS. Finally, in the second stage, the patient status and final outcome (discharge, morbidity, mortality) as well as the length of stay (LOS) were recorded retrospectively based on electronic documents provided by trained hospital staff.

### Statistical method

All data, including age, sex, trauma mechanism, KTS, eISS, and patient outcome, were entered into SPSS 27.0 (IBM Corp., Armonk, N.Y., USA) for analysis. Descriptive statistics were performed and The Mann–Whitney U-test was also used to compare the mean scores in the stratified groups. T-test was used to assess the difference of difference scores in discharge. The association of age and scores with LOS was analyzed with Pearson correlation. For mortality analysis, the hospitalized group was added to the discharge group. Logistic regression was used to analyze the level of correlation of scores with mortality, and produce Odds Ratios (OR) and risks. In our regression we included all the available variables. However, it should be pointed out that unlike KTS which has a discrete score for patient’s age and level of consciousness, eISS does not include these factors. Hence, we did not include age and level of consciousness as covariates in the analysis pertaining to KTS. The discernment accuracy of KTS and eISS was evaluated by the receiver operator characteristic (ROC) curve. The area under the curve (AUC) was calculated for each scoring system and compared using the Delong test in R package “pROC” version 1.18. Further comparison of the performance of KTS with eISS was made by sensitivity and specificity for mortality. The level of significance for all the analyses was set at *p* < 0.05.

## Results

### Baseline parameters

Of the patients who were admitted to the emergency department due to trauma, from December 2020 to March 2021, a total of 381 multi-trauma patients were included in the main analysis. During this study period, no traumatic patient with penetrating injuries was included in the study due to mismatching with the inclusion criteria or missing data or lack of patient’s consent. Table 1 summarizes the main results. The mean LOS was calculated for all the patients who were admitted to the hospital. (*n* = 147) However, 10 patients had conditions unrelated to trauma (e.g., tumor found incidentally in imaging studies) and were admitted to other wards after discharge from trauma ED. One could argue that some of these conditions were aggravated by the trauma. Hence, we decide to include both in our results. The “adjusted LOS” will refer to analysis done after removing these 10 patients. The mean LOS was 11.71 days while the adjusted LOS, had a mean value of 12.31.

**Table 1:** Different attributes of the patient population, based on the mechanism of trauma

Mechanism of Trauma		Car Crash	Fall	Motorcycle Crash	Bicycle Crash	Car to Pedestrian	Motorcycle to Pedestrian	Total
**Number**		159	97	67	7	44	7	381
**Gender (Male %)**		111(69.8%)	63(64.9%)	60 (89.6%)	5(71.4%)	34(77.3%)	6(85.7%)	279(73.2%)
**Age average (SD)**		32 (17)	44(24)	28(13)	17(3)	40(21)	42(24)	35.3 (19.7)
**eISS (Median (25th %- 75th %))**		11(4–19)	10(5–18)	14(5–19)	6(3–13)	10(5–14)	8(4–19)	10(5–18)
**KTS (Median (25th %- 75th %))**		13(12–15)	14(13–15)	14(12–15)	15(13–16)	14(13–15)	15(13–15)	14(12–15)
**GCS (Median (25th %- 75th %))**		13(10–15)	14(12–15)	13(10–15)	15(13–15)	13(12–15)	15(11–15)	13(11–15)
**LOS (Median (25th %- 75th %))**		10(6–18)	6(3–13)	6(4–9)	6(4–9)	9(5–13)	6(5–6)	8(5–13)
**Adjusted LOS (Median (25th %- 75th %))**		10(6–18)	6(3–13)	6(5–10)	7(5–10)	11(7–13)	6(5–6)	9(5-14.5)
**Outcome**	**Mortality**	24(15.1%)	21(21.6%)	15(22.4%)	3(42.9%)	4(9.1%)	1(14.3%)	65(17.1%)
	**hospitalization**	85(53.5%)	54(55.7%)	38(56.7%)	4(57.1%)	27(61.4%)	4(57.1%)	211(55.4%)
	**Discharge**	50(31.4%)	22(22.7%)	14(20.9%)	0(0%)	13(29.5%)	2(28.6%)	105(27.6%)

The in-hospital mortality rate (death in inpatient wards and the emergency department) was 17.1%. 27.6% of patients did not need admission and after initial therapeutic interventions in the emergency department, were discharged and the remainder were admitted to trauma wards. The age difference between trauma groups was significant (p value < 10^− 5^) but the Kruskal-Wallis test for difference in outcome between trauma types were not significant (p value = 0.078). Mann-Whitney U test was significant for the difference in outcome between genders; male patients had higher chances of mortality and hospitalization (p value = 0.017).

The score differences between male and female patients were not statistically significant in GCS and KTS (P value = 0.24 and 0.13 respectively) but significant for eISS (P value = 0.008). Age was not correlated to GCS and eISS (p value = 0.978 and 0.554 respectively).

### Length of stay

Overall, the LOS between different types of trauma was not significant (p value = 0.166). However, post hoc tests using Least Significant Difference (LSD) only showed significance between the car crash and motorcycle crash groups, with the former requiring longer hospitalizations (p value = 0.014). The age of the patients showed a weak correlation with LOS (*r* = 0.21, p value = 0.011) and gender did not affect the LOS (p value = 0.33). Patients’ GCS at the time of admission showed a moderate correlation with the LOS (*r*=-0.39, p value < 10^− 5^). KTS and eISS both showed a considerable correlation with LOS, however, KTS was a stronger predictor (*r*=-0.527, p value < 10^− 5^ and *r* = 0.462, p value < 10^− 5^).

Using adjusted data for LOS, the analysis did not yield different results and only slight changes in p values were observed. The difference between types of trauma had a p value of 0.252 and difference between car crash and motorcycle group had a p value of 0.024. Pearson correlation analysis of “adjusted LOS” for age, GCS, KTS, and eISS were *r* = 0.19 (p value = 0.025), *r*=-0.37 (p value < 10^− 5^), *r*=-0.51 (p value < 10^− 5^) and *r* = 0.43 (p value < 10^− 5^), respectively.

### Mortality prediction

The difference in mortality rates between different types of trauma was not significant. (p value = 0.234) As expected, the age of the patients had a significant correlation with mortality, showing a 2.1% (95%CI: 0.8–3.4) increased risk with each added year of age (p value = 0.002).

The results of the logistic regression analysis for KTS indicated that lower Kampala trauma score was a significant predictor of mortality (p value < 10^− 5^), with a decrease in the score being associated with an increase in the odds of mortality (OR 1.301; 95% CI: 1.211–1.397). However, gender was not a significant predictor of mortality (*p* = 0.909).

In regression analysis for eISS, eISS score was positively associated with mortality, where for each unit increase in the score, the odds of mortality increased by 16.5% (OR: 1.165, 95%CI: 1.097–1.236, p value < 10^− 5^). Conversely, Glasgow coma scale was negatively associated with mortality, where for each unit increase in the score, the odds of mortality decreased by 42.2% (OR: 0.578, 95%CI: 0.486–0.688, p value < 10^− 5^). Additionally, Age was positively associated with mortality, where for each year increase in age, the odds of mortality increased by 3.7% (OR: 1.037, 95%CI: 1.014–1.061, *p* = 0.002). Gender and Type of Trauma were not found to have a significant association with mortality outcome (Gender: OR = 0.818, 95%CI: 0.298–2.248, *p* = 0.697; Type of Trauma: OR = 1.188, 95%CI: 0.945–1.492, *p* = 0.140).

### Comparing two methods

**Fig. 1:**
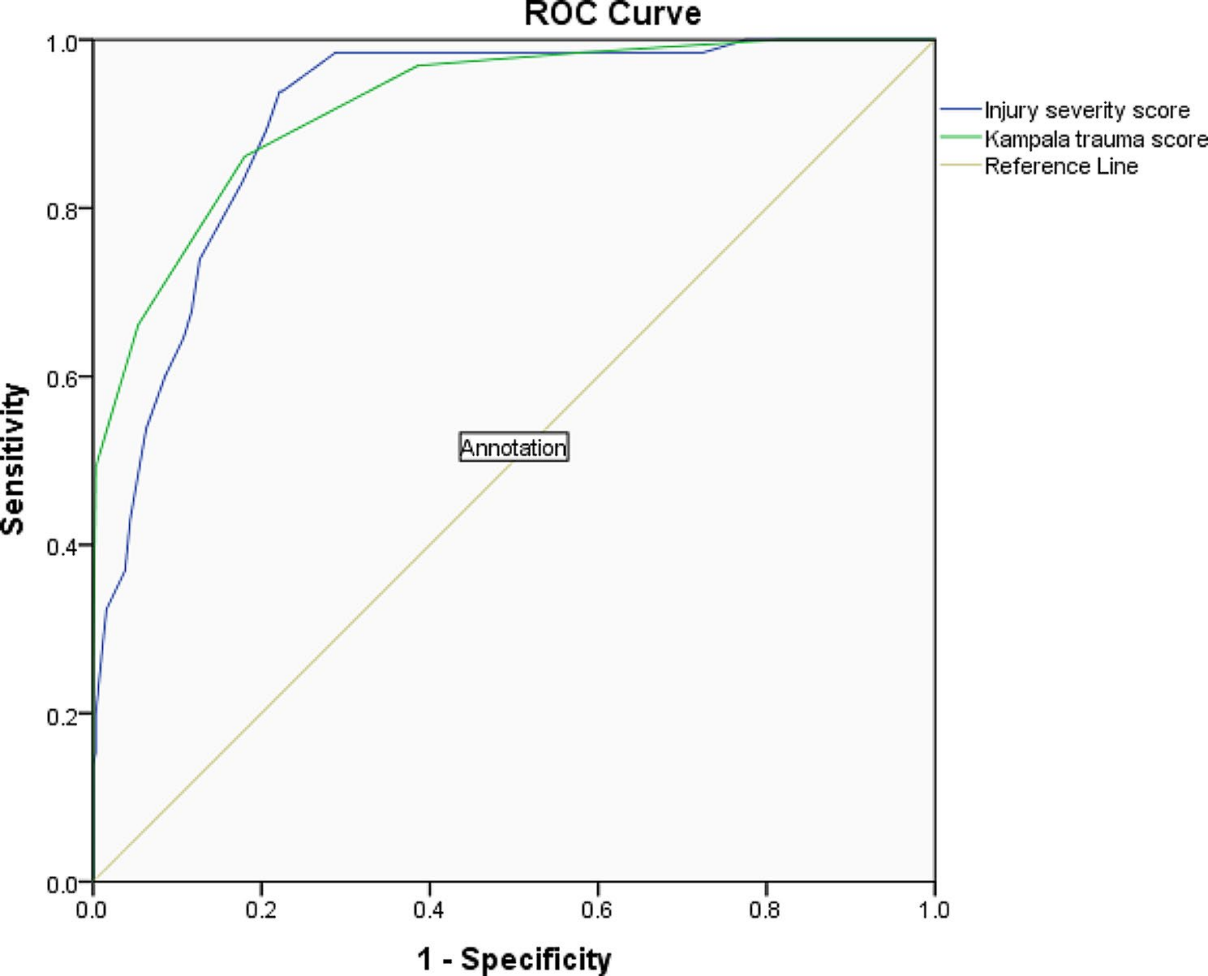
Comparison of ROC curves of KTS and eISS as predictors of in-hospital mortality for all presenting traumas

Figure 1 illustrates the ROC curve for each of the eISS and the KTS as predictors of mortality. The AUC for KTS was 0.923 (95%CI: 0.888–0.958) and for eISS was 0.910 (95% CI: 0.877–0.944). In comparing the AUCs by the Delong test, the difference between areas was 95%CI: -0.0145-0.0404 and it was not statistically significant (*p* = 0.356).

A cut-off of 12.5 in KTS (at this point, the sensitivity and the specificity are 87.7% and 82%, respectively) and a cut-off of 16.5 in eISS (at this point, the sensitivity and the specificity are 83.1% and 79.4%, respectively) were optimal to predict mortality. In order to make the study more practical in clinical settings, a cut-off of below 13 for KTS and above 16 for eISS was assigned. The diagnostic odds (DOR) ratio for KTS and eISS were 28.27 (95%CI = 13.22–60.45) and 32.00 (95%CI = 13.95–73.40) respectively.

Due to the low number of cases in some trauma types, analyzing the subgroups did not yield meaningful results.

## Discussion

In order to determine the possibility of replacing a valid and feasible trauma scoring system and simplifying mortality prediction in a resource-limited setting, we conducted a study to assess the predictive accuracy of KTS in the outcome of multi-traumatic patients compared to eISS.

Based on the ROC curves in our study, the AUC of the KTS (0.923) was higher than that of the eISS (0.910), however, this was not significant. Therefore, we can assume the ability of KTS to discriminate mortality was on par with eISS. Although the sensitivity and specificity of the two tests were briefly compared, the ROC curve is a more effective indicator than others [15]. The high AUC results in our study, may be due to the higher quality of care provided at the study hospital compared to other LMICs. The most important shortcomings of our ED are limited personnel considering the high load of patients and limited access to high-tech diagnostic and therapeutic modalities. However, during certain times of day (or months of year) with low or moderate patient load, these limitations have diminished impact.

According to the previous study correlation between scores and the severity of the injury led to an overlap of confidence intervals [28]. Due to the high correlation between KTS and eISS, and high mortality and morbidity rates, the overlap of AUC confidence intervals should not be interpreted as a study limitation [29]. 

Our findings are consistent with studies conducted in other developing countries [19, 20]. The prospective multicenter study in India found that the ability of both KTS and RTS to predict mortality was better than that of ISS or NISS [24]. The multi-hospital study in Kenya showed, considering in-hospital mortality, both KTS and TRISS had better discrimination than eISS [19]. Also, as we found, previous studies have shown that estimated ISS (eISS) as a prospective trauma registry can be used effectively to promote injury management policy [17, 18]. 

It is noteworthy that the study conducted on data collected from an American level-1 trauma registry found that the discrimination of the KTS approached that of TRISS and outperformed both ISS and RTS [30]. Results of the previous study showed that the favorable discernment of KTS in other studies should not be simply considered the poor performance of ISS in LMICs [30]. In developed countries, due to optimal pre-hospital care, intubation, and sedation of injured patients prior to hospital arrival, a KTS-based scoring system (modified KTS (M-KTS)) is often compared to other injury scoring systems. Several studies indicated that the M-KTS is a robust predictor of mortality in trauma patients throughout the United States [9]. A recent review study concluded that KTS is a tool to strengthen trauma systems in LMICs and also suggested it for trauma systems that do not measure trauma severity in high-income countries [31]. 

In line with previous studies, we found that older patients were more likely to be hospitalized longer [4, 32]. We also found that an increase in the severity of the injury is associated with prolonged LOS and confirmed a strong correlation between lower KTS and prolonged LOS. In addition, the current study showed that trauma patients who did not die in the early days of admission and had a prolonged LOS demonstrated a higher ability to survive [33]. 

Given that the eISS and KTS are calculated without the need for retrospective review, both are easier to implement than western utilized scores (ISS, TRISS, RTS) [17, 23, 34]. Implementing these scoring systems in clinical practice requires consideration of various factors, such as availability of data, training of healthcare providers, and integration into electronic health records. For example, the KTS score can be calculated quickly and easily using only basic clinical information, such as the patient’s consciousness level and respiratory rate. In contrast, the eISS score requires more detailed information about the nature and extent of the injury, which may require more advanced imaging or diagnostic procedures. Additionally, healthcare providers may require specific training to accurately calculate and interpret these scores.

This study has several limitations as it is a single-center analysis of multi-trauma patients. First, it was not possible to benchmark and compare therapeutic interventions and medical care among other trauma centers. The second limitation of this hospital-based study is that trauma patients who died before reaching the hospital were not involved in the analysis due to unavailable information. These limitations can be resolved in future studies by conducting a multi-center study and also expanding the study to pre-hospital surveillance. For future research, we recommend a similar study on triage levels 2 and 3 with a larger sample size.

Third, considering various methods for calculating the number of serious injuries, evaluating KTS among different studies may be challenging. However, Gardner et al. found that KTS had a clinically significant ability to predict the need for hospitalization regardless of whether a serious injury was ascertained by physician judgment or estimated AIS score [35]. Fourth, although factors such as underlying disease, urbanization, and insurance coverage can also have an effect on the LOS and outcome, we have not considered these factors in our analysis and interpretations [36–38]. Fifth, the limited time frame of the study might have skewed the results pertaining to the injury mechanisms. Due to extreme cold in the winter in the region, most outdoor activities are limited and lower crime rates are reported in winter. This subsequently results in rarely having penetrating injury that are not related to traffic accidents or falls. To overcome this limitation, future studies should be conducted in warm seasons or the full one-year period. Sixth, as we mentioned, due to the small number of patients in some trauma type subgroupings, the subgroup analysis did not have significant results. This limitation can be overcome in future studies with the cooperation of other trauma centers and increasing the sample size.

Furthermore, we did not register patients who did not consent to participate in this study or had one of the defined exclusion criteria. Due to our ethic guidelines, upon ascertainment of exclusion criteria we were not allowed to gather any further information from that moment onward. Therefore, it was not possible to perform missing case analysis in this study. This limitation primarily requires more lenient ethical regulations.

As in any resource-constrained setting, having a prediction system for better care planning and resource allocation is paramount. We believe that KTS could be one of the rings of such a chain to manage resource allocation in tertiary and secondary centers. Further utility of these scoring systems can be explored in the future, with more robust studies, regarding the exact parameters and available (or unavailable) resources that make a setting differentially more suitable to implement one or the other measure.

According to our study, in settings with limited resources, adaptations (eISS) or alternative scoring systems (KTS) could be appropriate in assessing the injury. To precisely identify the most severe cases, system faults, and preventable deaths, further research should concentrate on determining the ideal scoring system across stages of trauma under resource constraints.

In the present study, patients who were the driver or passenger of a car accounted for the largest proportion of injured people, and highest hospitalization duration. Among other countries around the world, Iran has a high mortality rate of RTC [39, 40]. Scoring systems can be considered useful tools in improving quality of care for these patients.

In high income nations, trauma scoring system are frequently used to guide agendas for research and quality improvement [41]. Trauma Quality Improvement Programs (TQIP) use various metrics in their evaluations, and reliable metrics to compare observed and predicted mortality in patients is one such metric [42]. These scores can be used to benchmark trauma center outcomes against regional and national averages which would allow them to compare and improve their processes and patient outcome [42, 43]. This also enables comparing effectiveness of hospital-specific protocols and identifying areas that require more resources and attention of the hospital management [42, 43]. 

However, it should be noted that these metrics fail to identify a some of the avoidable deaths and miss important opportunities for system improvement and using these scores alone may not be sufficient to improve quality of care, since other factors also play a crucial role in improving trauma care. These factors include provider expertise, resource availability, and system-level factors, such as pre-hospital care and transport, trauma team activation. Hence, these scores should be used in conjunction with other quality improvement initiatives.

Inadequate funding, absence of standards for clinical documentation and management, and a shortage of permanent staff allocated to emergency wards are problems in LMICs’ trauma care systems [44, 45]. Iran’s pre-hospital emergency services are relatively far from international standards in the majority of domains [46]. The Kenyan government is also obviously required to develop methods and solutions that consider the difficulties of organizing and standardizing treatment in pre-hospital settings [44]. Moreover, Kenya lacked organizational capacities, such as trauma registries, trauma-specific training, and quality improvement plans [47]. . As in Kenya, concerns about the front-line medical team’s education and training standards remain unresolved in Iran [48]. 

There is the absence of organized and integrated systems of trauma care in both Iran and India [48–50]. Less than 7% of injured patients in India were transported by ambulance; however, up to 40% of trauma patients in Iran used emergency medical vehicles for transferring to the hospital [50]. The mean total response time for all the UMICs, including Iran, was less than 60 min, whereas this time in LMICs, such as India, was measured in hours rather than minutes [50]. In countries without a well-established trauma team, in-hospital trauma care protocol, or trauma surgeon, such as Iran and India, death by injury accounted for a higher portion of total deaths [50]. Both India and Iran had insufficient trauma centers, which were not logically distributed between cities and rural regions [50]. Trauma centers were located in large cities due to financial limitations and inadequate health infrastructure [51]. 

As injuries are recognized as a major cause of morbidity and mortality in developing countries such as Iran, research in this context is required to identify opportunities for prevention and enhanced treatment, as well as to determine priorities and the distribution of limited resources [20, 52]. In order to establish performance improvement programs to guide improvements in patient care, it will also be essential to have simple and readily available scoring systems in such settings [20]. However, we did not measure the resource utilization in implementing these scores in our study, and this would be an important area for future research.

About half of all trauma-related deaths (including patients who die in the early hours of admission, often due to severe head, chest, or abdominal trauma (30%), and those who die usually due to multi-organ failure or sepsis at a later time of hospitalization (20%)) can be reduced by a fast and efficient therapeutic strategy [15]. A coordinated approach to assessing the severity of injury helps guide therapeutic and improve the quality of care provided to trauma patients [53]. Despite the lack of sufficient financial resources, specialized manpower, and advanced diagnosis capacity, which are significant limitations to achieving more accurate ISS assessments, the most commonly used score of injury severity remains ISS in LMIC settings such as Iran [5, 8, 23]. 

## Conclusion

In this patient population, the KTS has similar accuracy in predicting the mortality of multi-trauma patients compared to the eISS. We also confirmed a strong correlation between lower KTS and prolonged length of stay. Both of these scoring systems can be used for the early evaluation of patients in ED for the prediction of patient outcomes, depending on available resources, physicians’ experience and judgment, and hospital guidelines.

### Electronic supplementary material

Below is the link to the electronic supplementary material.

Supplementary Material 1

## Data Availability

Data is provided within the supplementary file.

## Abbreviations

ISS: Injury Severity Score
LMIC: Low- and Middle-Income Countries
KTS: Kampala Trauma Score
eISS: Estimated Injury Severity Score
GCS: Glasgow Coma Scale
AUC: Area Under the Curve
HIC: High-Income Countries
AIS: Abbreviated Injury Scale
LOS: Length Of Hospital Stay
EMS: Emergency Medical Services
AVPU scale: Alert, Voice, Pain, Unresponsive
FAST: Focused Assessment with Sonography in Trauma
MRI: Magnetic Resonance Imaging
SD: Standard Deviation
ED: Emergency Department
LSD: Least Significant Difference
CI: Confidence Interval
ROC: Receiver Operating Characteristic
DOR: Diagnostic Odds Ratio
RTC: Road Traffic Crashes
TQIP: Trauma Quality Improvement Programs
TRISS: Trauma and Injury Severity Score
RTS: Revised Trauma Score
